# Supplementary material for: Assessing Prevalence and Unique Risk Factors of Suicidal Ideation among First-Year University Students in China Using a Unique Multidimensional University Personality Inventor
Source: Int J Environ Res Public Health. 2022 Aug 30;19(17):10786. doi: 10.3390/ijerph191710786 (PMC9517881; doi:10.3390/ijerph191710786)
Supplement: Supplementary file 1 [file ijerph-19-10786-s001.zip › ijerph-1835851-supplementary Table S1.pdf]

University personality inventory is a 60-item self-report measure assessing whether an individual usually experienced the described symptom during the past year. For each item, a score of 1 was given for “Yes”, and 0 was given for “No”. Lie scales include items 5, 20, 35, and 50, which check the positive aspects of the student's character. Students with a UPI total sum score above 20 or those who respond “yes” to item 25 (“Have an idea of wanting to die”) are identified and guided to arrange personal interviews with mental health professionals

University personality inventory

1	Poor appetite
2	Feel sick, stomachache
3	Easily have diarrhea or constipation
4	Care about palpitation and pulse
6	Full of dissatisfaction and complaints
7	High expectation from parents
8	My past and family is misfortune
9	Over-worry about my future
10	Do not like meeting others
11	Feel that I am not myself
12	Lack of enthusiasm and positivity
13	Pessimistic
14	Distracted
15	Over-uneven in emotion
16	Frequent insomnia
17	Headache
18	Ache in neck and shoulder
19	Chest pain or feel oppressed
21	Intolerance
22	Inclined to worry
23	Restless
24	Irritable
25	Have idea of wanting to die
26	No interest in anything
27	Declining memory
28	Lack of patience
29	Lack of judgment
30	Too dependent on others
31	Distressed by blushing
32	Stuttering, faltering voice
33	Feel hot and cold
34	Concern about urination or sexual organs
36	Uneasy without reason
37	Feel uneasy when alone

38	Lack of confidence
39	Irresolute about anything
40	Easily feel misunderstood
41	Lack faith in others
42	Over-suspicious
43	Unwilling to associate with others
44	Feel self-abased
45	Catastrophizing
46	Physically exhausted
47	In cold sweat when I hurry
48	Dizzy when I stand up
49	Have ever lost consciousness, cramp
51	Over-rigid
52	Cannot give up repeating things
53	Susceptible to dirtiness
54	Cannot get rid of meaningless idea
55	Sense weird smell from myself
56	Suspect others say something bad about me
57	Wary of others
58	Care about others' gaze
59	Feel others despise me
60	Sensitive emotions
